# Nanographene-Based Polymeric Nanoparticles as Near-Infrared Emissive Neuronal Tracers

**DOI:** 10.1021/acsnano.4c10754

**Published:** 2024-12-13

**Authors:** Hao Zhao, Laurent Guillaud, Maria Fransiska Emily, Xiushang Xu, Liliia Moshniaha, Hiroki Hanayama, Ryota Kabe, Marco Terenzio, Akimitsu Narita

**Affiliations:** † Organic and Carbon Nanomaterials Unit, Okinawa Institute of Science and Technology Graduate University, 1919-1 Tancha, Onna-son, Kunigami-gun, Okinawa 904-0495, Japan; ‡ Molecular Neuroscience Unit, Okinawa Institute of Science and Technology Graduate University, 1919-1 Tancha, Onna-son, Kunigami-gun, Okinawa 904-0495, Japan; § Max Planck Institute for Polymer Research, Ackermannweg 10, 55128 Mainz, Germany; ∥ Organic Optoelectronics Unit, Okinawa Institute of Science and Technology Graduate University, 1919-1 Tancha, Onna-son, Kunigami-gun, Okinawa 904-0495, Japan

**Keywords:** nanographene, near-infrared emission, neuronal tracer, retrograde axonal transport, neuroscience

## Abstract

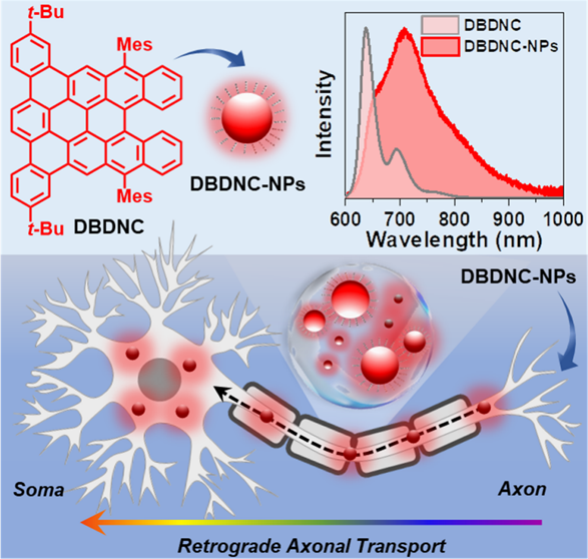

Precise tracking of axonal transport is key to deciphering neuronal functions. To achieve long-term imaging at both ultrastructural and macroscopic resolutions, it is critical to develop fluorescent transport tracers with high photostability and biocompatibility. Herein, we report the investigation of nanographene (NG)-based polymeric nanoparticles (NPs) as near-infrared (NIR)-emissive neuronal tracers. Dibenzo[*a*,*m*]dinaphtho[3,2,1-*ef*:1′,2′,3′-*hi*]coronene (DBDNC) was employed as the NG, which exhibited a broad NIR emission with a maximum at 711 nm inside the NPs. DBDNC-NPs displayed high photostability and low cytotoxicity, enabling live tracing of retrograde axonal transport in mouse sensory neurons cultured in microfluidic chambers. We also elucidated how DBDNC-NPs undergo retrograde axonal transport following the endolysosomal pathway. This work provides a proof of concept for NIR-emissive, NG-based neuronal tracers with potential for applications in neurobiology.

Visualization of axonal projections plays a crucial role in the mapping of brain connectivity at both ultrastructural and macroscopic resolutions.^1−3^ Key to visualizing neuronal anatomical connections is the development of appropriate fluorescent neuronal tracers that can be endocytosed and transported retrogradely (from axon to soma) or anterogradely (from soma to axon).^4,5^ While viruses^6−8^ and inorganic quantum dots^9−12^ are commonly used in neuroscience as neuronal tracers, other materials, such as synthetic dextrans,^13,14^ latex nanoparticles,^15−17^ and small organic fluorophores,^18^ have also been investigated. However, existing tracers often have drawbacks, including biological hazard, toxicity, and fading fluorescence emission, which are not compatible with long-term tracing studies in living cells or *in vivo*.^19^ On the other hand, near-infrared (NIR) fluorescence can potentially increase the imaging resolution, circumventing tissue autofluorescence and enabling deep tissue penetration.^20−22^ A few NIR luminescent materials have been used for neural cell and/or tissue imaging,^23−25^ but NIR-emissive tracers have rarely been explored.^15^

Polymeric nanoparticles (NPs), fabricated by encapsulating organic dyes inside amphiphilic polymers, typically through the nano-reprecipitation method, exhibit clear advantages over synthetic fluorophores and other nanomaterials, such as ease of preparation and the possibility of the surface functionalization for various bioapplications.^26−29^ For instance, NPs have been applied to phototheranostics of bacterial infections and cancer,^30−35^ drug and gene delivery,^36−38^ as well as artificial photosynthesis.^39−41^ Nevertheless, such polymeric NPs have seldom been considered for applications in neuroscience.^42^

Large polycyclic aromatic hydrocarbons, also called “nanographenes” (NGs), can be synthesized bottom-up with atomically precise graphene-like nanostructures. NGs exhibit structure-dependent optical and electronic properties as well as chemical and photostability, attracting increasing attention as next-generation carbon nanomaterials.^43−46^ Biological applications of NGs have unfortunately been hampered by a lack of water solubility.^47,48^ In this work, we report neuronal tracers based on water-dispersible polymeric NPs containing NGs, which displayed a broad NIR emission extending to 1000 nm (Figure 1). These NPs showed high photostability and low cytotoxicity, enabling real-time monitoring of their axonal uptake and retrograde transport associated with endolysosomes along microtubules in living neurons.

**Figure 1:**
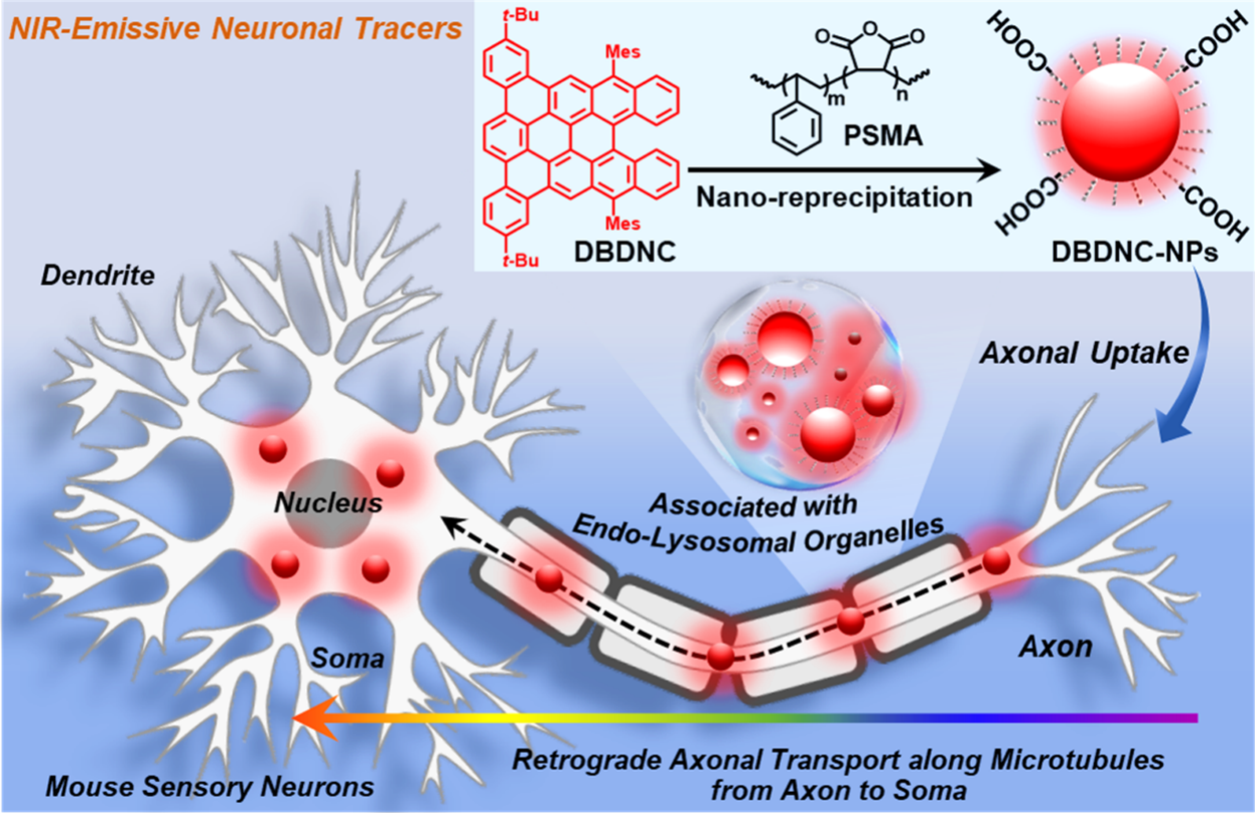
Schematic illustration of the preparation of DBDNC-NPs as NIR-emissive neuronal tracers and a proof-of-concept experiment in this work to study live tracing of retrograde axonal transport using mouse sensory neurons.

## Results and Discussion

**Figure 2:**
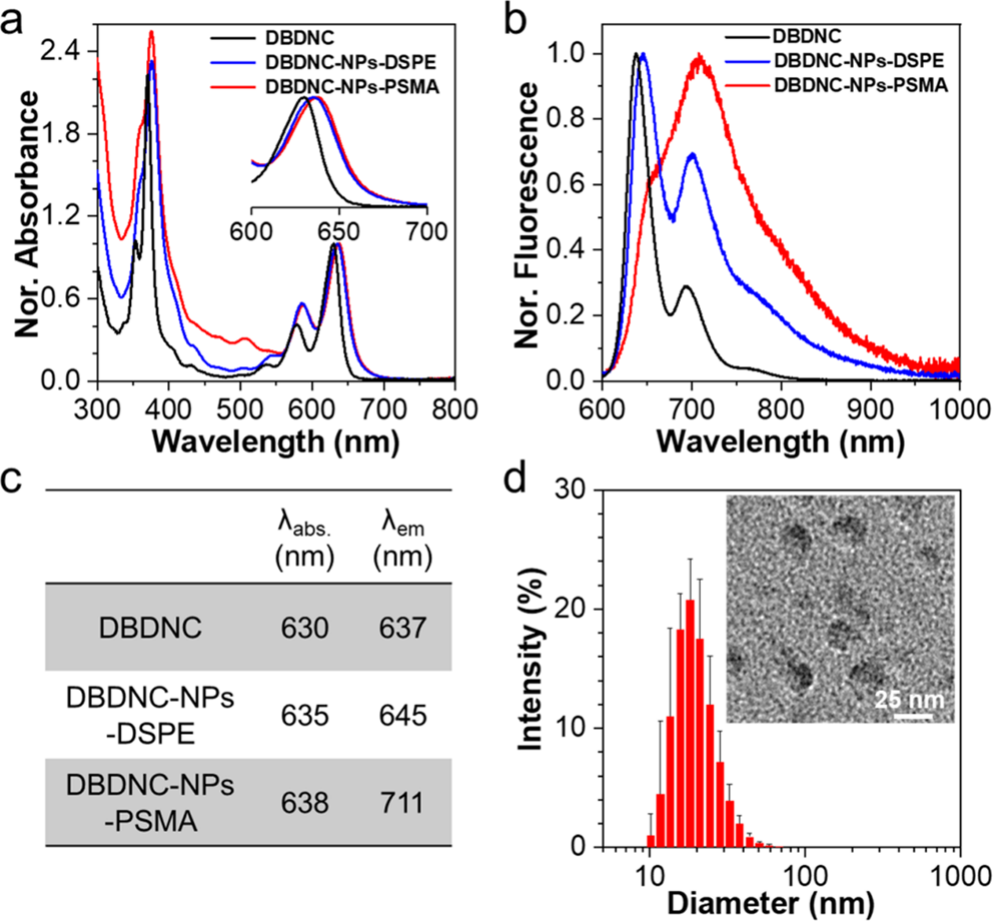
Characterization of DBDNC-NPs. (a) Normalized absorption and (b) fluorescence spectra of DBDNC (8 μg mL^–1^) in THF, compared with DBDNC-NPs-PSMA and DBDNC-NPs-DSPE in water (DBDNC: 25 μg mL^–1^; polymer: 50 μg mL^–1^). λ_ex_ = 580 nm. (c) Summary of the absorption and emission maxima. (d) Size distribution and TEM images (inset) of DBDNC-NPs. Scale bar: 25 nm.

We selected dibenzo[*a*,*m*]dinaphtho[3,2,1-*ef*:1′,2′,3′-*hi*]coronene (DBDNC) as a highly photostable NG with strong red emission^49^ and fabricated DBDNC-based polymeric NPs (DBDNC-NPs) through a nano-reprecipitation method^32,50−52^ by adding a solution of DBDNC molecules and poly(styrene-*co*-maleic anhydride) (PSMA; see Figure 1 and the Methods section for chemical structure and more details, respectively) in tetrahydrofuran (THF) into water followed by removal of THF (Figure 1; see the Methods section for details). PSMA is commonly used as an amphiphilic polymer for the fabrication of polymeric nanoparticles.^53−55^ The phenyl groups of the polystyrene moieties can promote the efficient encapsulation of hydrophobic organic dyes and fluorescent polymers, which are typically based on aromatic structures.^56,57^ DBDNC-NPs-PSMA showed the longest-wavelength absorption peak (λ_abs_) at 638 nm and a broad NIR emission at 711 nm with a tail extending to ∼1000 nm (Figure 2a–c). Notably, the fluorescence spectrum of DBDNC-NPs-PSMA is distinct from that of DBDNC in THF with the emission maxima at 637 nm, presumably due to the local concentration of the DBDNC molecules inside the NPs, reminiscent of aggregation-induced red shift reported for organic dye-based polymeric NPs.^58,59^ The aggregation effect in DBDNC-NPs-PSMA was corroborated by gradually decreasing the DBDNC/PSMA mass ratio from 0.5:1 to 0.05:1, showing varying ratios of the fluorescence intensities at 637 and 711 nm (Figures S1–S3). In contrast, DBDNC-NPs were prepared using 1,2-distearoyl-*sn*-glycero-3-phosphoethanolamine-*N*-[carboxy(polyethylene glycol)-2000] (DSPE-PEG2000-COOH; see Figure S4 and the Methods section for chemical structure and more details, respectively) displayed less significant changes in the emission spectrum, compared to DBDNC-NPs-PSMA prepared with the same mass ratio, although their longest-wavelength absorption bands and photoluminescence quantum yields were very similar (Figure 2a–c and Table S1). Additionally, the fluorescence lifetime of DBDNC-NPs-PSMA was measured to be ∼2–4 ns (Figures S5–S7) while that of DBDNC-NPs-DSPE was ∼7 ns (Figure S8), closer to the value of 8.5 ns previously obtained for DBDNC molecules in solution.^49^ These results indicate the possibility of modulating the optical properties of polymeric NPs by selecting different amphiphilic polymers, causing more significant aggregation of encapsulated molecules with PSMA as a random copolymer compared to that with DSPE-PEG2000-COOH as a block copolymer in the current case. Aggregation effects inside NPs have been investigated typically by changing the molecular structure and concentration,^58,60^ but our findings provide a rare insight into the effect of different amphiphilic polymers on the aggregation state of encapsulated molecules.^61,62^

DBDNC-NPs-PSMA with a DBDNC/PSMA mass ratio of 0.5:1 was selected for subsequent bioevaluations, considering their intense NIR-emissive properties, and simply called DBDNC-NPs below. Dynamic light scattering (DLS) analysis of DBDNC-NPs revealed an average diameter of 20 ± 2 nm with polydispersity index (PDI) of 0.22 (Figure 2d), in agreement with their sizes observed by transmission electron microscopy (TEM) (Figures 2d and S9). The ζ-potential of DBDNC-NPs was also measured to be −40.8 ± 1.6 mV, indicating a negative surface charge consistent with the presence of carboxyl groups (Figure S10).^52,63^ The carboxyl groups can allow for further surface conjugation with recognition moieties, contributing to the cell-selective and targeting bioimaging applications.^53−55^ The DLS, ζ-potential, and TEM characterizations of the other DBDNC-based NPs provided comparable results (Figures S1–S3 and S11).

**Figure 3:**
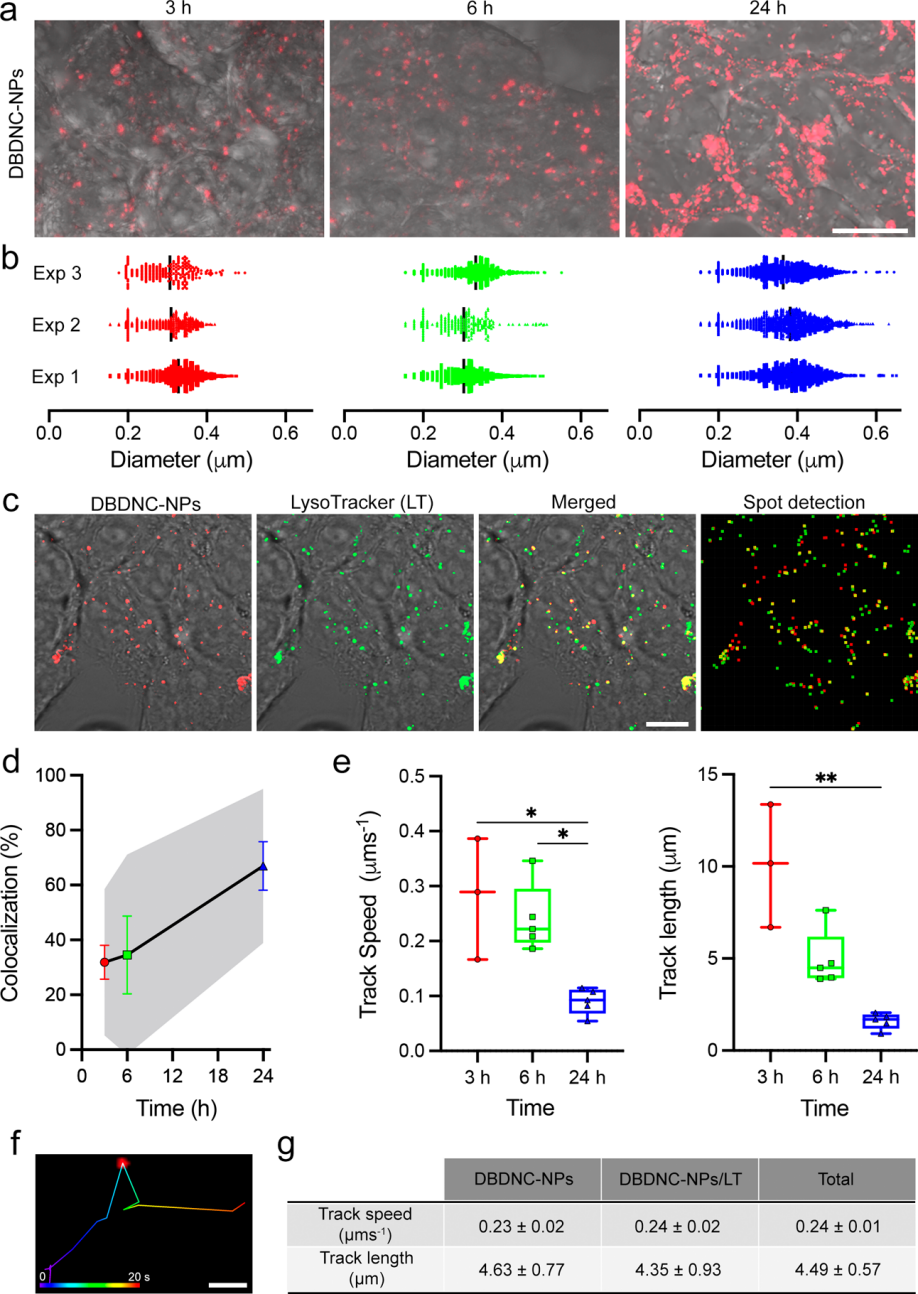
Cellular uptake, intracellular distribution, and mobility of DBDNC-NPs in HEK293T cells. (a) Uptake of DBDNC-NPs and (b) size distributions of DBDNC-NPs-positive organelles from 3 experiments (experiments 1–3) in HEK293T cells at various time intervals. Scale bar: 20 μm. (c) CLSM and the corresponding spot center detection images of HEK293T cells labeled with DBDNC-NPs and LT. Scale bar: 20 μm. (d) Percentage of colocalization of DBDNC-NPs with LT after 3 h (red), 6 h (green), and 24 h (blue) of incubation. Data are presented as mean ± s.e.m. and 95% confidence interval (CI; shaded area; *n* = 3–5 experimental replicates). (e) Track speed and track length of DBDNC-NPs after 3 h (red), 6 h (green), and 24 h (blue) of incubation. Data are presented as boxplot (median with min and max whiskers, *n* = 3–5 experimental replicates), Kruskal–Wallis with Dunn’s multiple comparison test, *p = 0.042 and 0.036, **p = 0.006. (f) Tracing of a single DBDNC-NPs-positive organelle in HEK293T cell cytoplasm. Trace was color-coded according to time (blue = 0 s, red = 20 s). Scale bar: 1 μm. (g) Summarized track speed and track length of DBDNC-NPs alone or associated with lysosomes (DBDNC-NPs/LT), data shown as mean ± s.e.m.

In order to reduce the number of mice required for the isolation of primary neurons, optimal experimental conditions for cellular uptake, intracellular distribution, and mobility of DBDNC-NPs were explored in human embryonic kidney 293T (HEK293T) cells, which are commonly used to study intracellular trafficking.^64−66^ The internalization of DBDNC-NPs in HEK293T cells was revealed by high-resolution confocal laser scanning microscopy (CLSM), as evidenced by the accumulation of intracellular signal from DBDNC-NPs and the increase in the size distribution of DBDNC-NPs-positive organelles (Figures 3a,b, and S12 and Video S1). In addition, DBDNC-NPs colocalized with LysoTracker (LT) (Figure 3c and Video S2), in a way that was proportional to the incubation time (Figure 3d), suggesting that DBDNC-NPs might follow the endocytic pathway from early to late endosomes/lysosomes.^67,68^ The mobility of DBDNC-NPs-positive organelles decreased significantly at 24 h postloading compared to 3 h, as shown by the reduction in track speed and length (Figure 3e), in line with their progressive entry into the endocytic pathway and eventual accumulation in lysosomes.^67−69^

Based on the aforementioned data regarding intracellular mobility and colocalization with lysosomes, we chose 6 h postloading to further characterize the trafficking properties of DBDNC-NPs. An autoregressive motion algorithm analysis^11^ was performed to track the movement of single DBDNC-NPs in cell cytoplasm (Figure 3f), revealing an average track length of 4.49 ± 0.57 μm with a speed of 0.24 ± 0.01 μm s^–1^ for the total population of DBDNC-NPs (Figure 3g). Moreover, the track length of the pool of DBDNC-NPs non-colocalizing with lysosomes (4.63 ± 0.77 μm) was more pronounced than the ones associated with them (4.35 ± 0.93 μm). The track speed was, however, similar between the two populations. Our data on the speed (0.24 ± 0.02 μm s^–1^) and size distribution (∼0.3 μm) of DBDNC-NPs-positive organelles in HEK293T at 6 h postloading is in line with the speed (∼0.4 μm s^–1^) and size (∼0.3 μm) of lysosomes in previous reports.^70,71^

**Figure 4:**
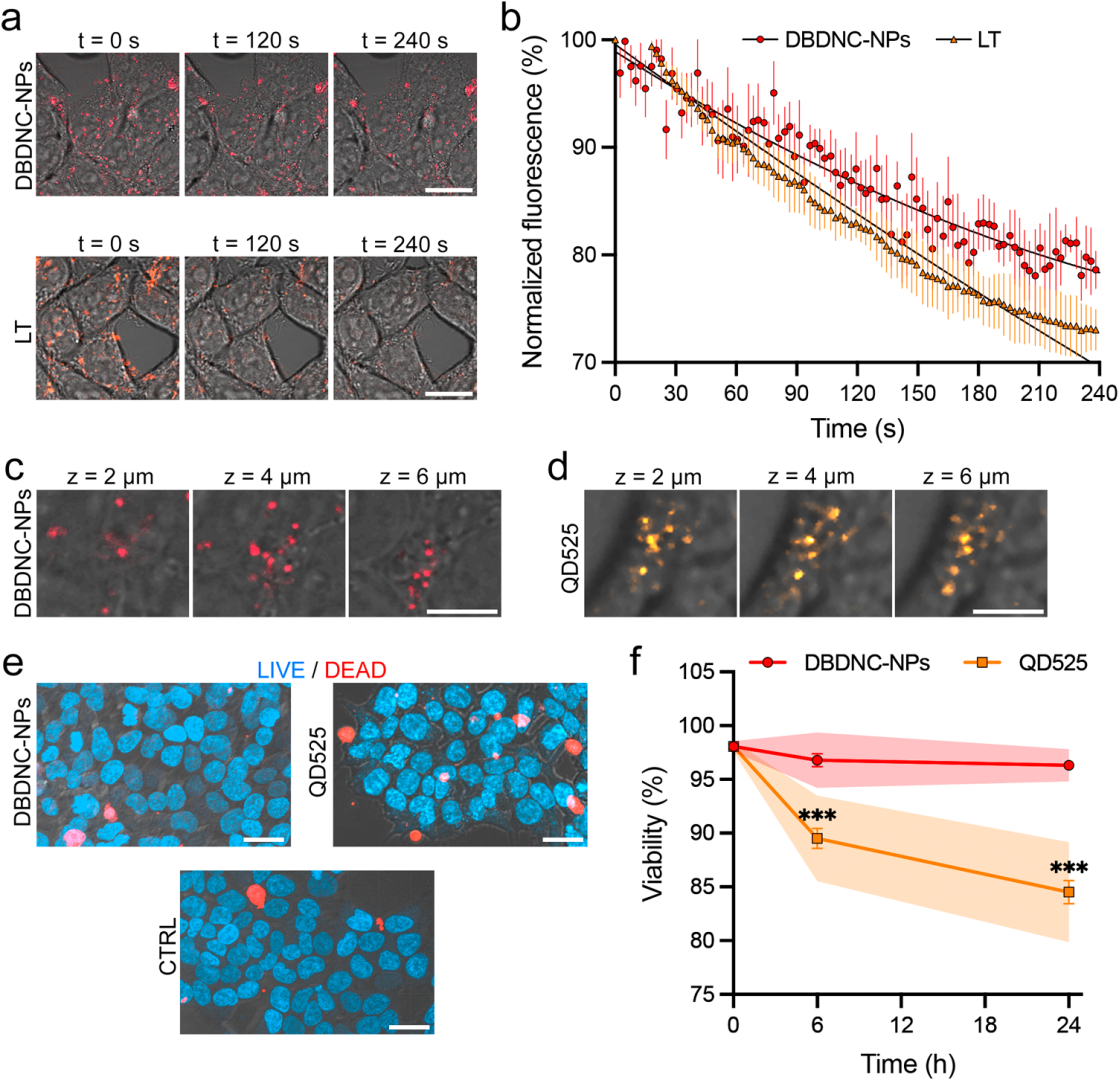
Evaluations of photostability and biocompatibility of DBDNC-NPs. (a) CLSM images of HEK293T cells incubated with DBDNC-NPs and LT upon scanning for 0, 120, and 240 s and the corresponding (b) normalized fluorescence intensity profiles of DBDNC-NPs (red) and LT (orange). Data are shown as mean ± s.e.m. (*n* = 3 experimental replicates). Scale bar: 20 μm. CLSM Z-stacks images (2–6 μm) of HEK293T cells at 6 h postloading of (c) DBDNC-NPs or (d) QD525. Scale bar: 5 μm. (e) CLSM images of HEK293T cells stained with Nucblue and Cytopainter red at 24 h postloading of DBDNC-NPs or QD525. Scale bar: 20 μm. (f) Viability of HEK293T cells at 6 and 24 h postloading of DBDNC-NPs (red) or QD525 (orange). Data shown as mean ± s.e.m. and 95% CI (shaded area; *n* = 3 experimental replicates), multiple unpaired *t*-test with Welch correction, ***p = 0.0046.

Photostability and biocompatibility of the DBDNC-NPs were then investigated. In contrast to the reduced intracellular fluorescence of LT, DBDNC-NPs retained significantly stronger signals at the end of the acquisition (Figure 4a). Quantification analysis verified a lower intensity loss of DBDNC-NPs (<20%) compared to LT (>20%), highlighting the higher photostability of the former. The higher photostability of DBDNC-NPs can be ascribed to aromatic stabilization of NGs like DBDNC, without having vulnerable unsaturated chains or heterocycles.^47,49,72^ In addition, encapsulation in such NPs can also suppress the photobleaching of DBDNC molecules by inhibiting the reaction with reactive oxygen species generated through photosensitization.^73^ Biocompatibility of DBDNC-NPs was next evaluated by a Live/Dead assay in comparison to that of inorganic CdSe quantum dots (QD525), which are widely used as markers of intracellular trafficking in neuroscience. The internalizations of DBDNC-NPs and QD525 were quantified in HEK293T cells after 6 h-incubation (Figure 4c,d). More cells were positive for Cytopainter red, indicating their dead state upon incubation with QD525 compared to that of HEK293T cells incubated with DBDNC-NPs (Figure 4e). Indeed, DBDNC-NPs (5.4 μM, calculated based on the used concentration of 5.0 μg mL^–1^ and molecular weight of DBDNC molecules without PSMA) showed negligible cytotoxicity in HEK293T cells, while QDs 525 with the concentration of 8 nM significantly reduced cell viability by ∼10 to ∼15% (Figure 4f). These results are in accordance with previous toxicity data for CdSe QDs^74,75^ and demonstrate the significantly lower cytotoxicity of DBDNC-NPs.

NIR-emissive DBDNC-NPs were further used to label living neurons to evaluate their potential as neuronal tracers. Two-layered microfluidic chambers (MFC) featuring two main chambers connected with microgrooves/channels were fabricated using poly(dimethylsiloxane) (PDMS)^76^ (see also the Methods section for the details). These MFCs were used to culture mouse sensory primary neurons for 14 days. A net flow from soma toward the axon side, which allows for the fluidic separation of neuronal cell bodies from their axo-terminals, was achieved by adding a larger volume of medium to the soma chamber.^76,77^ To confirm the fluidic isolation of the device, both the soma and axonal chambers were incubated with anti-β3-tubulin primary antibody, while the corresponding Alexa Fluorophore (AF) 568-conjugated secondary antibody was added to the axonal chamber only. As expected, imaging revealed that only the axonal network was stained (Figure S13). As a control, AF488-conjugated secondary antibody was added to the soma chamber, resulting in the staining of both soma and axonal network, due to the net flow from the soma chamber to the axonal one created by the aforementioned disparity of volumes, which is the driving force behind the fluidic isolation of the axonal chamber (Figure S13).

**Figure 5:**
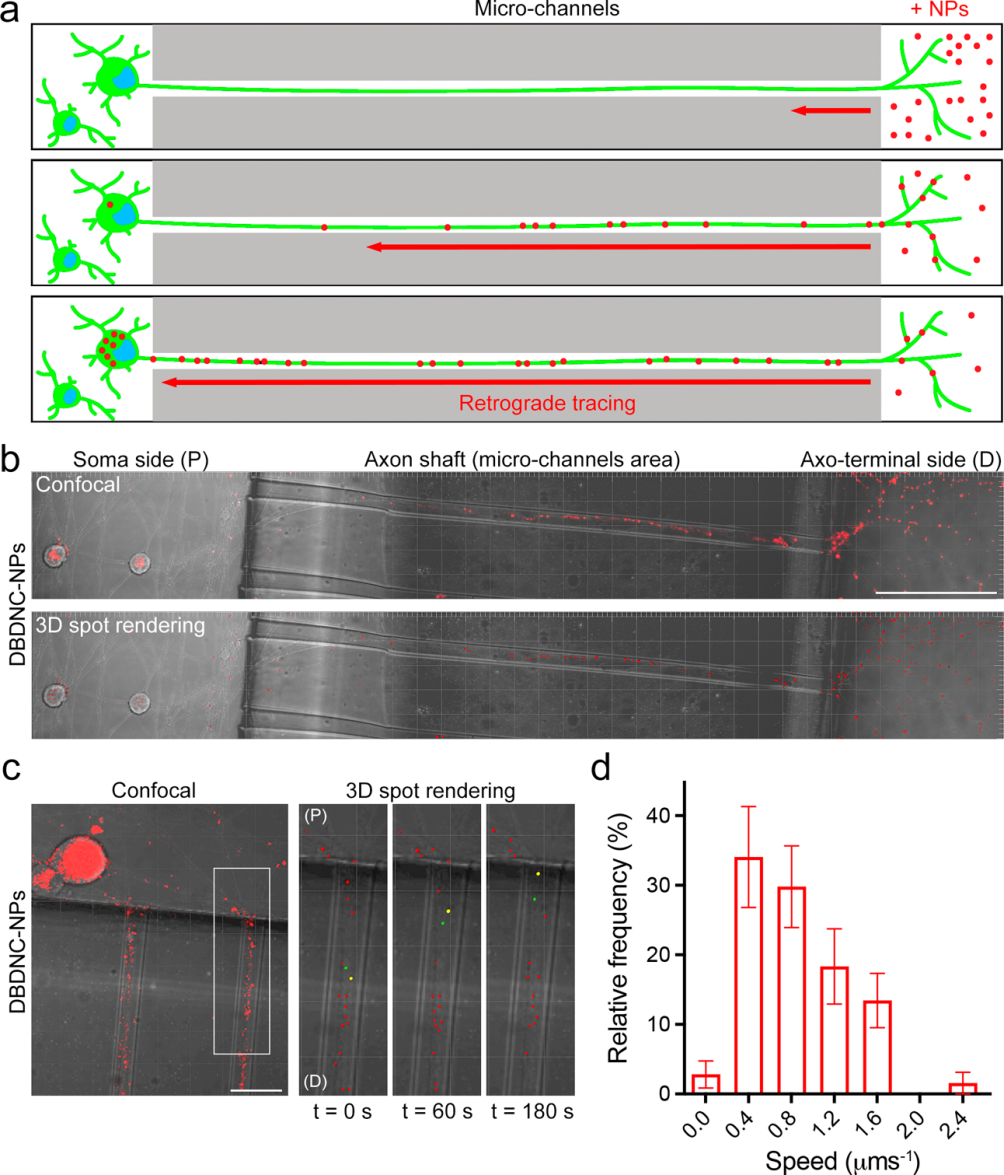
Transport of DBDNC-NPs in live primary neurons cultured in a two-layered microfluidic chamber. (a) Schematic illustration of the MFC experimental setup. (b) Live CLSM and 3D spot rendering images of the retrograde transport of DBDNC-NPs along the axonal shaft to the neuronal soma. Scale bar: 100 μm. (c) CLSM images of DBDNC-NPs-positive organelles (yellow and green spots) during the retrograde movements and (d) the corresponding speed distributions. “Time = 0 s” is the start of the image acquisition. Scale bar: 20 μm. (P): proximal area (soma side) and (D): distal area (axo-terminal side). Data are shown as mean ± s.e.m. (*n* = 17 NPs from 3 MFC cultures obtained from 3 mice).

DBDNC-NPs were then added only to the axonal chamber (Figure 5a), to test for the retrograde transport from the axons to the soma and evaluate their potential as a neuronal tracer. Live CLSM showed successful endocytosis of DBDNC-NPs in axons (D, axo-terminal side), followed by retrograde transport along the axon shafts to the neuronal soma (P, soma side) (Figure 5b and Videos S3 and S4). Notably, the majority of DBDNC-NPs were transported retrogradely from axon to soma with a speed ranging from 0.4 to 1.6 μm s^–1^ (Figure 5c,d, and Video S5), which was slightly different from the detected track speed of DBDNC-NPs in HEK293T cells (0.24 ± 0.02 μm s^–1^), presumably due to the cell-type-dependent cell membrane lipid composition and endocytic/transport mechanisms.^78,79^ Moreover, the retrograde transport of DBDNC-NPs from the axonal to the soma side in the MFC-cultured neurons was further confirmed by the increasing number of DBDNC-NPs accumulating in the neuronal soma as a function of incubation time (Figure S14).

**Figure 6:**
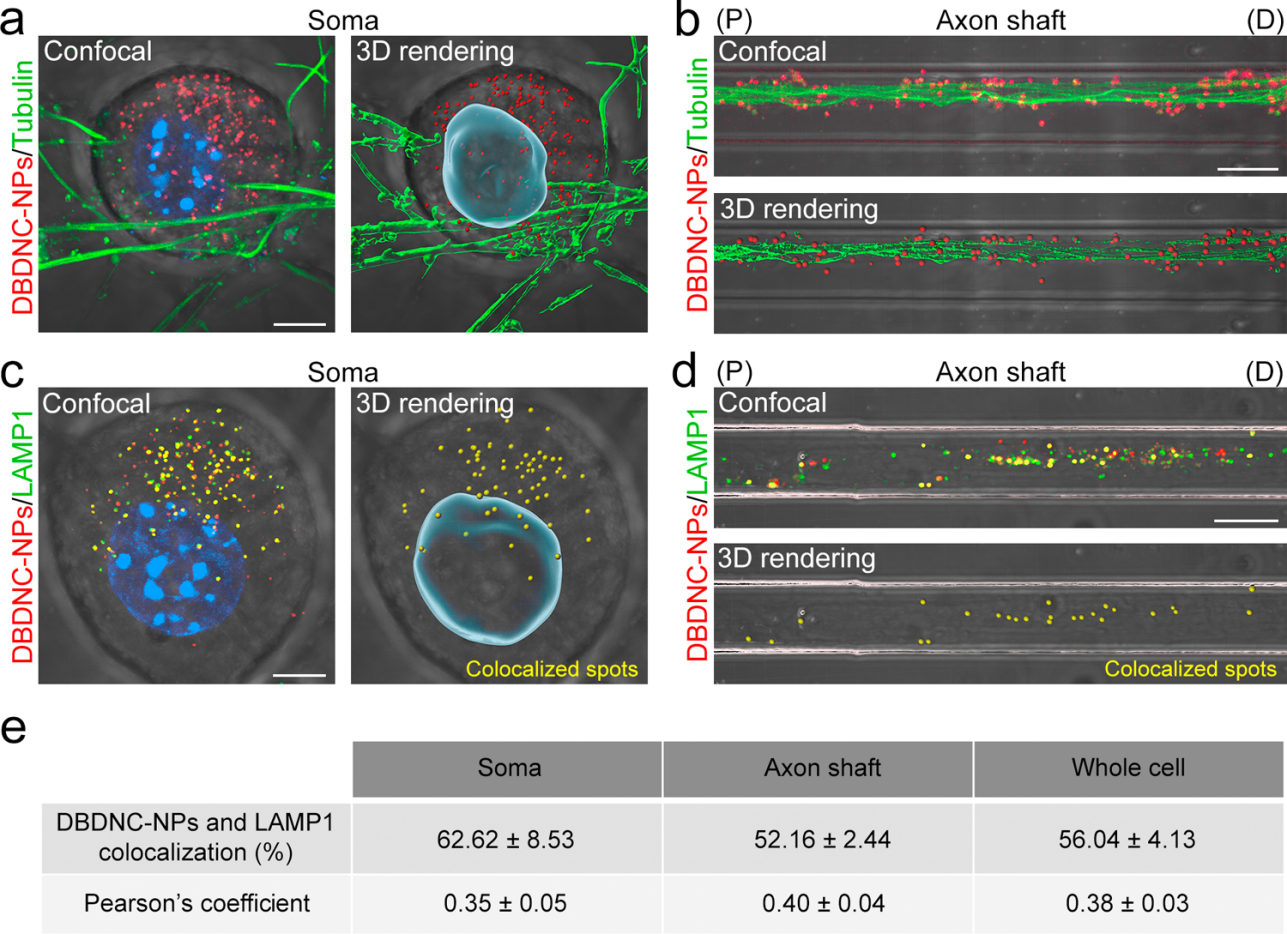
Transport mechanism of DBDNC-NPs in primary neurons. Live CLSM and 3D rendering images of neuronal soma and axons labeled with DBDNC-NPs and (a, b) tubulin-tracker (microtubule marker) or (c, d) LAMP1 (lysosome marker) in MFC culture, and cell nucleus labeled with Nucblue. Scale bar: 5 and 10 μm for the images of soma and axons, respectively. (e) Summarized colocalization and Person’s coefficient of DBDNC-NPs colocalization with lysosomes (DBDNC-NPs/LAMP1). Data shown as mean ± s.e.m. (*n* = 8 (soma) and 12 (axons) from 3 MFC cultures obtained from 3 mice).

The retrograde transport mechanism of DBDNC-NPs in primary neurons was investigated by colocalization analysis with tubulin (microtubule marker) and lysosomal-associated membrane protein 1 (LAMP1, an endolysosomal marker). Accumulation of DBDNC-NPs was observed in the soma and axon shaft, where they were tightly localized along the microtubule (Figure 6a,b). We also confirmed the colocalization of DBDNC-NPs with LAMP1 after accumulation in the soma (colocalization = 62.62 ± 8.53%, Pearson’s coefficient = 0.35 ± 0.05, Figure 6c,e) and axon shaft (colocalization = 52.16 ± 2.44%, Pearson’s coefficient = 0.40 ± 0.04, Figure 6d,e), suggesting that DBDNC-NPs were retrogradely trafficked to the soma after cellular uptake through the endolysosomal pathway along microtubules, as >50% is already a high degree of colocalization in neuroscience in comparison with the significant values of ∼30–50% in the literature.^80−82^

## Conclusions

In summary, we developed nanographene-based polymeric NPs as NIR-emissive neuronal tracers, expanding the potential biological applications of nanographenes to neuroscience. The dispersion of DBDNC as fluorescent nanographene into aqueous media was realized through its assembly with amphiphilic polymers to form nanoparticles with hydrophilic surfaces (DBDNC-NPs), which displayed a broad NIR emission. DBDNC-NPs demonstrated high photostability and low cytotoxicity, enabling real-time tracing of retrograde axonal transport associated with endolysosomes along microtubules in live neurons. These NIR-emissive NPs are, thus, compatible with other conventional fluorescent neuronal tracers for the visualization of axonal projections and potentially for mapping brain circuits in multichannels. With the ability to be retrogradely transported and the feasibility of surface functionalization using the already existing carboxyl groups, DBDNC-NPs can potentially be exploited to further our understanding of axonal transport, including its implications in disease.^83−86^ Thus, we believe that DBDNC-NPs can be a powerful tool, which expands the toolkit available to neuroscientists.

## Methods

### Materials

All chemicals were used as received without further purification. The synthesis of DBDNC was previously reported.^49^ Cumene terminated PSMA with 75 wt % of styrene and an average *M*_n_ of ∼1900, as determined by gel permeation chromatograph, was purchased from Sigma-Aldrich (#442402). DSPE-PEG2000-COOH with the chemical formula C_131_H_257_NNaO_55_P and molecular weight of 2780.38 g mol^–1^ (average values due to polydispersity of poly(ethylene glycol)) was obtained from Merck (Avanti Polar Lipids 880135P, powder). QD525 was purchased from Thermo Fisher. Dulbecco’s modified Eagle’s medium (DMEM), fetal bovine serum (FBS), and phosphate buffer saline (PBS) were obtained from Wako, Thermo Fisher, and GibcoBRL, respectively. LysoTracker Green, Nucblue, and Cytopainter red were provided by Thermo Fisher and Abcam, respectively. Anti-β3-tubulin antibody was commercially available from Synaptic System. Anti-LAMP1 monoclonal antibody conjugated with AlexaFluor-488 was commercially available from Thermo Fisher. Deionized (DI) water was obtained from a Milli-Q system (Millipore).

### Measurements

UV–vis absorption spectra were recorded on a UV–vis–NIR spectrophotometer (Shimadzu UV-3600 Plus). Fluorescence spectra were recorded on a Fluorescence + absorbance spectrometer (Horiba Duetta). Fluorescence decays were recorded using a streak camera system (C14832-110, Hamamatsu Photonics) equipped with a 300 mm spectrograph (SpectraPro, HRS-300-SS, Princeton Instruments). The excitation was provided by a Yb:KGW femtosecond laser (PHAROS, Light Conversion) with an optical parametric amplifier (ORPHEUS, Light Conversion). CLSM images were taken on a confocal laser scanning microscope (LSM900 confocal microscope (Carl Zeiss GmbH)) equipped with an on-stage incubation chamber (Pecon). Size and ζ-potential measurements were performed using a Nano ZS 90 (Malvern, U.K.) instrument equipped with a He–Ne laser (633 nm, 4 mW). TEM images were taken on a transmission electron microscope (ThermoFisher Titan G2).

### Preparation of DBDNC-NPs

DBDNC-NPs were prepared through nano-reprecipitation method.^50^ 0.25 or 0.1 or 0.025 mg of DBDNC molecules and 0.5 mg of PSMA were dissolved in 5 mL of THF to form a homogeneous solution. The solution was then quickly added into 15 mL of DI water under sonication, which was stirred at room temperature with continuous nitrogen bubbling for 48 h to remove THF and produce DBDNC-NPs-PSMA with different mass ratios of DBDNC to PSMA, namely, 0.05:1, 0.2:1, and 0.5:1, which were specified as DBDNC-NPs-PSMA (0.05:1), DBDNC-NPs-PSMA (0.2:1), and DBDNC-NPs-PSMA (0.5:1), respectively. The obtained DBDNC-NPs dispersions were concentrated to 1 mL by centrifugal filter units (100 kDa, Millipore) at 1500 rpm for 5 min and then diluted with DI water to 10 mL, followed by concentration to 1 mL again by the centrifugation. This procedure was repeated three times in total. The obtained stock dispersions were stored in the dark at 4 °C. DBDNC-NPs with the amphiphilic polymer of DSPE-PEG2000-COOH with the mass ratios of DBDNC to DSPE-PEG2000-COOH of 0.5:1 (0.25 mg:0.5 mg) were also prepared using the method above, which was named DBDNC-NPs-DSPE (0.5:1).

### Fluorescence Lifetime Measurements

The laser pulse width was 165 fs, and the frequency was 25 Hz. The 580 nm wavelength was used for excitation, and integration was made for a range of 600–825 nm for all samples. The fluorescence lifetimes were determined by fitting the two-phase exponential decay function with a fixed time offset and *x*_0_ by eq 1
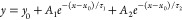
1where *y*_0_ is offset, *x*_0_ is center, *A*_1_ and *A*_2_ is amplitude, and τ_1_ and τ_2_ is time constant.

### PLQY Measurements

PLQY values were measured using an integrating sphere with a photoluminescence measurement unit (Quantaurus-QY, C11347-01, Hamamatsu Photonics). The water dispersions were deoxygenated by bubbling argon through the solutions (dispersions) for 15 min before the measurement. The 360 nm excitation light was used for the determination of QY for all samples.

### DLS Analysis of DBDNC-NPs

DLS measurements were carried out in a polystyrene cuvette at a 173° accumulation angle after equilibrating for 2 min at 25 °C. The data were processed by the instrument software (Zetasizer Nano software v3.30) to give the number mean particle size and polydispersity index value by the non-negative least-squares method. ζ-Potential of DBDNC-NPs was measured by a Malvern Zetasizer Nano ZS 90 instrument simultaneously.

### TEM Analysis of DBDNC-NPs

A 10 μL aliquot of DBDNC-NPs dispersion with a concentration of 25 μg mL^–1^ was deposited on a microgrid precoated with ultrathin film (UHR-C10, Okenshoji Co., Ltd.). After 5 min, excess dispersion was removed by soaking with filter paper. Then, the microgrid was dried under reduced vacuum for 3 h before TEM observation, which was operated at 80 kV.

### Culture of HEK293T Cells

HEK293T cells were maintained in DMEM low glucose (Wako #041-29775) supplemented with 10% FBS (Thermo Fisher #10270106) in 10 cm cell culture dishes (Violamo #VTC-D100). HEK293T cells were then plated into 35 mm glass-bottom culture dishes (ibidi no. 81158) for further experiments.

### DBDNC-NPs Loading in HEK293T Cells

DBDNC-NPs were diluted into culture medium at a final concentration of 5 μg mL^–1^, which were added gently to culture dishes containing HEK293T. Cultures were then processed for live or fixed CLSM imaging after 3, 6, and 24 h postloading.

### Labeling Lysosomes with DBDNC-NPs in HEK293T Cells

Labeling lysosomes in live HEK293T cells was performed with LT (LysoTracker Green DND-26, Thermofisher #L7526) for colocalization with DBDNC-NPs. LT was used at a final concentration of 75 nM according to the instructions from the manufacturers. LT was directly added to cell culture medium and cells were incubated for 30–45 min at 37 °C and 5% CO_2_ before live CLSM imaging.

### QD525 Loading in HEK293T Cells

QD525 (Invitrogen) were diluted into culture medium at a final concentration of 8 nM and added gently to the culture dishes containing HEK293T cells. Cultures were then processed for live or fixed confocal imaging after 3, 6, and 24 h postloading.

### Cell Viability of HEK293T Cells

Toxicity of DBDNC-NPs and QD525 was measured on HEK293T cells using live/dead fluorescent labeling with Cytopainter cell viability assay kit (Fluorometric-Red) (Abcam #ab176744). Briefly, cell culture medium was removed and cells were gently washed in warm Tyrode’s solution. 1 mL of warm Tyrode’s solution (Sigma-Aldrich #T2397-500ML) containing Cytopainter red (1/500 dilution) and Nucblue for live cells (1 drop for 500 mL, Thermo Fisher no. R37605) was gently added to the culture dish. Cells were incubated for 45 min at 37 °C and 5% CO_2_ before CLSM imaging. The percentage of cell viability was estimated by the ratio between the number of live cells (labeled with Nucblue) and the total number of cells.

### Fabrication of MFC

The fabrication of the microfluidic chamber for the culture of mouse sensory neurons was performed as previously described.^76^ The microfluidics device utilized in this study has 2 layers. The first layer consists of 2 main chambers that are connected by microchannels. One chamber is dedicated to the seeding of the neurons and to fluidically isolate their cell bodies from the other chamber containing the axons and terminals. The second layer consists of 4 reservoirs, each located at the opening of the chambers. The device was fabricated using PDMS and was bonded to a glass-bottom Petri dish using plasma bonding. The device was sterilized using 70% ethanol and coated with poly-l-lysine 1 day prior to neuronal culture. On the day of the culture, poly-l-lysine was washed out, and laminin was added to the chambers for 1 h.

### Primary Dissociated Culture of Mouse Sensory Neurons in MFC

Dorsal root ganglia neurons were extracted from 8- to 10-week-old mice and dissociated as previously described.^77^ Experiments involving mice have been performed in accordance with the regulations of the OIST animal care and use committee (protocol #2023-001). OIST animal facilities and animal care and use program are accredited by the Association for Assessment and Accreditation of Laboratory Animal Care (AAALAC) International (reference #1551). Neuronal cell suspension was injected into the cell body chamber on the first layer. The device was allowed to sit for 5 min to make the cell settle to the glass bottom before the F-12 growth medium was added to the reservoir. To maintain fluidic isolation, a larger volume of medium (120 μL) was added in the cell body reservoir and a lower volume (100 μL) in the axon side reservoir to establish a net flow from the soma side toward the axon side. Neurite growth factor (NGF) was then added to the axonal chamber to control the directional growth of the axon toward the axonal chamber. Neurons were grown inside an incubator at 37 °C and 5% CO_2_ for 14 days before performing experiments. After 14 days in culture, axons have already crossed to the axonal side and form a dense axonal network.

### Testing Fluidic Isolation by Immunofluorescence

Neurons cultured on MFC were fixed in PBS with 4% PFA for 30 min at room temperature. Samples were further permeabilized and blocked in PBS solution containing 0.3% Triton-X and 5% goat serum (GS) for 30 min. β3-tubulin antibody (Synaptic System #302304) was diluted (1/500) in PBS containing 0.03% Triton-X and 0.5% GS and incubated overnight at 4 °C. After 3 washes in PBS, fluorescent secondary antibodies AlexaFluor-488 (ThermoFisher #A11073) and AlexaFluor-568 (ThermoFisher #21435) were diluted (1/500) in PBS containing 0.03% Triton-X and 0.5% GS. 120 μL of AlexaFluor-488 was added to the soma side reservoir, and 100 μL of AlexaFluor-568 was added to the axon side reservoir. Secondary antibodies were incubated for 1 h at room temperature. After 3 washes in PBS, samples were ready for image acquisition.

### Loading of DBDNC-NPs in MFC Culture

DBDNC-NPs were diluted to 5 mg mL^–1^ in 100 μL of F-12 medium and gently added into the axo-terminal chamber of the microfluidic device. 120 μL of F-12 medium without NPs was added into the soma side of the microfluidic device. DBDNC-NPs were incubated for 6–24 h before washing out and replacing with fresh F-12 medium followed by image acquisition.

### Labeling of Microtubules in Living Neurons Cultured in MFC

After loading DBDNC-NPs, microtubules were labeled with tubulin-tracker green for 30 min at 37 °C and 5% CO_2_ according to the manufacturer’s instructions (ThermoFisher #T34075). Tubulin tracker was added to both chambers. Nucblue Live (Thermo Fisher #R37605) was added (1 drop for 500 μL of F-12 medium) to the chamber containing cell bodies for labeling the nucleus, followed by the addition of fresh F-12 medium prior image acquisition.

### Immunostaining of Lysosomes in Neurons Cultured in MFC

After loading of DBDNC-NPs, neurons cultured in MFC were fixed with 4% paraformaldehyde solution in PBS for 30 min at room temperature. Samples were further permeabilized and blocked with a PBS solution containing 0.3% Triton-X and 5% GS for 30 min. Anti-LAMP1 monoclonal antibody conjugated with AlexaFluor-488 (Thermo Fisher #MA5-18121) was diluted (1/500, v/v) in PBS containing 0.03% Triton-X and 0.5% GS, which was used to incubate the samples for 1 h at room temperature. After three wash cycles, PBS containing Nucblue for fixed samples (Thermo Fisher #37606) was added to label the nucleus.

### CLSM Imaging

Confocal laser scanning microscopy was performed on an LSM900 confocal microscope (Carl Zeiss GmbH) equipped with on-stage incubation chamber P-set 2000 (Pecon #133-800 261) at 37 °C and 5% CO_2_, using a plan-apochromat 63× DIC M27 oil-immersion objective (NA = 1.4, Carl Zeiss GmbH). For live imaging, acquisition parameters were adjusted to maintain a frame time of approximately 1.2 s (initial image parameters: 512 × 512 pixels resolution with 2.06 ms pixel dwell time, bidirectional scan without averaging, and the pinhole diameter was set to 1 au). Excitation laser line (2% laser power) was 561 nm for DBDNC-NPs with emission filters set to 656–700 nm. Nucblue was imaged with standard DAPI excitation/emission settings (405/410–514 nm) and 1% laser power. LysoTracker Green and TubulinTracker Green were imaged with excitation/emission at 488/400–550 nm and 0.2–0.5% laser power. QD525 were imaged with excitation/emission at 405/400–560 nm and 2% laser power. Cytopainter red was imaged with standard Alexa-568 excitation/emission settings (561/595–700 nm) and 0.2% laser power. Culture medium from HEK293T cells and mouse sensory neuron cultures was replaced with Tyrode’s solution (Sigma-Aldrich #T2397-500ML) before live imaging. Single plane time series were acquired while focus was automatically maintained throughout the acquisition period using Definite Focus 2.0 system (Carl Zeiss GmbH). Confocal Z-stacks time series were acquired without Definite Focus, with 0.5–1 μm stacks. Imaging of fixed samples loaded with DBDNC-NPs and immuno-labeled with LAMP1 was performed with the following excitation/emission settings: 561/650–700 nm at 2% laser power, 488/400–555 nm at 0.2–05% laser power and 405/410–514 nm at 3% laser power for DBDNC-NPs, LAMP1 and Nucblue, respectively.

### CLSM Imaging Analysis

Detection and tracking analyses of DBDNC-NPs and LT were performed using Imaris 9.9 software with Imaris Track and Measurement Pro Plugins (Bitplane Oxford Instruments). The autoregressive motion algorithm analysis was performed with an initial spot size of 200 nm with background subtraction, and an estimated maximum distance between 2 consecutive spots of 1–1.5 μm was used. DBDNC-NPs were also tracked manually using the Manual Tracking plugin in Fiji imaging software (https://imagej.net/software/fiji/). Percentage of colocalization, Pearson’s coefficient, and dynamic properties data such as track speed and length, displacement, and size were automatically calculated in Imaris Measurement Pro plugin and exported to Prism 10 (GraphPad Software, Inc.) for statistical analysis.

### Statistical Analysis

No statistical method was used to predetermine the sample size. Experiments were not randomized, and investigators were not blinded to allocation during the experiments. Each experiment was repeated a minimum of three times to ensure reproducibility and adequate statistical power. Statistical analysis and comparisons of data sets were performed in Prism 10 (GraphPad Software Inc.), and relevant statistical tests were added to the figure legends. Data are presented as mean ± s.e.m. or median value with minimum and maximum, and statistical significance (*) was assumed when *p* ≤ 0.05.

### Images and Figures Preparation

All CLSM images were acquired with ZEN Blue software (version 3.2, Carl Zeiss GmbH). Median or Gaussian filter was applied on CLSM images, and the filtered images were exported in TIFF image format. TIFF files were further processed in Photoshop 24.0 (Adobe) to create the final figures.

## Supporting Information Available

UV–vis absorption and emission spectra, DLS and ζ-potential data, TEM images, as well as the PLQY data and emission decays of the polymeric nanoparticles (PDF)
3D surface rendering of confocal Z-stacks of DBDNC-NPs in HEK293T cells (Video S1) (MOV)
Live confocal imaging of DBDNC-NPs (red) and lysotracker green (green) in HEK293T 6 h postloading (Video S2) (MOV)
Live confocal imaging of DBDNC-NPs (red) retrogradely transported from axon terminals along the axon to soma in MFC (Video S3) (MOV)
3D surface rendering of DBDNC-NPs (red) from Video S3 (Video S4) (MOV)
Zoom in view of live confocal imaging of DBDNC-NPs (red) retrograde transport in MFC (Video S5) (MOV)

## Supplementary Material

nn4c10754_si_001.pdf

nn4c10754_si_002.mov

nn4c10754_si_003.mov

nn4c10754_si_004.mov

nn4c10754_si_005.mov

nn4c10754_si_006.mov
