# Supplementary material for: Nanographene-Based Polymeric Nanoparticles as Near-Infrared Emissive Neuronal Tracers
Source: ACS Nano. 2024 Dec 13;18(51):34730–40. doi: 10.1021/acsnano.4c10754 (PMC11673580; doi:10.1021/acsnano.4c10754)
Supplement: Supplementary file 1 — nn4c10754_si_001.pdf [file nn4c10754_si_001.pdf]

Supporting Information

Nanographene-Based Polymeric Nanoparticles as Near-Infrared Emissive Neuronal Tracers

Hao Zhao,[†,‡] Laurent Guillaud,[◊,‡] Maria Fransiska Emily,[◊] Xiushang Xu,[†,§] Liliia Moshniaha,[ζ] Hiroki Hanayama,[†] Ryota Kabe,[ζ] Marco Terenzio[,◊], and Akimitsu Narita[*,†,§]*

[†]Organic and Carbon Nanomaterials Unit, Okinawa Institute of Science and Technology Graduate University, 1919-1 Tancha, Onna-son, Kunigami-gun, Okinawa 904-0495, Japan
[◊]Molecular Neuroscience Unit, Okinawa Institute of Science and Technology Graduate University, 1919-1 Tancha, Onna-son, Kunigami-gun, Okinawa 904-0495, Japan
[§]Max Planck Institute for Polymer Research, Ackermannweg 10, 55128 Mainz, Germany
[ζ] Organic Optoelectronics Unit, Okinawa Institute of Science and Technology Graduate University, 1919-1 Tancha, Onna-son, Kunigami-gun, Okinawa 904-0495, Japan
‡These authors contributed equally to this work

[*]E-mail: akimitsu.narita@oist.jp; marco.terenzio@oist.jp

Table of Contents

Supporting Data for the Polymeric Nanoparticles 3
Figure S1. 3
Figure S2. 3
Figure S3. 3
Figure S4. 4
Table S1........ 4
Figure S5. 4
Figure S6. 5
Figure S7. 5
Figure S8. 6
Figure S9. 6
Figure S10. 7
Figure S11. 7
Figure S12. 8
Figure S13. 8
Figure S14. 9

Supporting Data for the Polymeric Nanoparticles

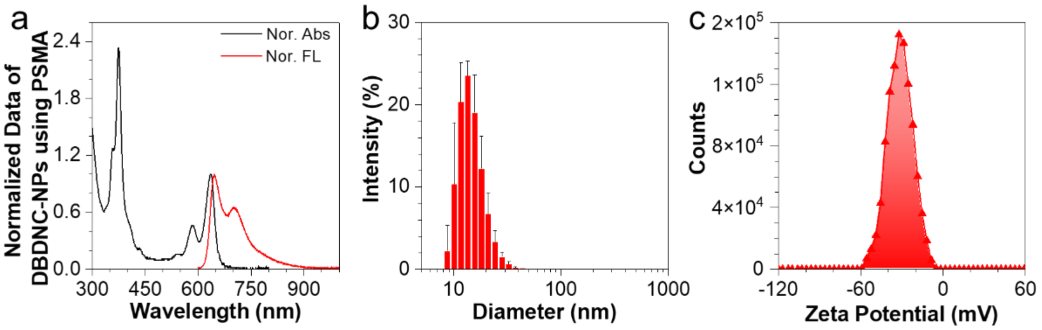

Figure S1. (a) Normalized absorption and fluorescence spectra of DBDNC-NPs-PSMA (0.05:1) in water. λ_{ex} = 580 nm for recording emission spectrum. (b) Size distribution of DBDNC-NPs-PSMA (0.05:1) by DLS analysis. (c) Zeta potential of DBDNC-NPs-PSMA (0.05:1) in water. The concentration of NPs was 2.5 μg mL^{-1}, based on the weight of DBDNC without PSMA.

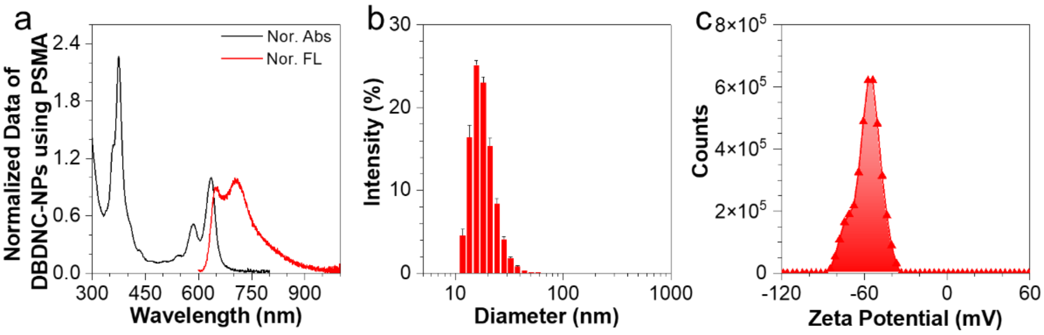

Figure S2. (a) Normalized absorption and fluorescence spectra of DBDNC-NPs-PSMA (0.2:1) in water. λ_{ex} = 580 nm for recording emission spectrum. (b) Size distribution of DBDNC-NPs-PSMA (0.2:1) by DLS analysis. (c) Zeta potential of DBDNC-NPs-PSMA (0.2:1) in water. The concentration of NPs was 10 μg mL^{-1}, based on the weight of DBDNC without PSMA.

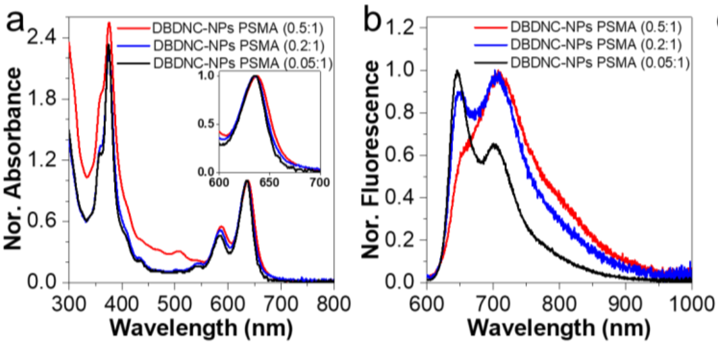

c

	$\lambda_{abs.}$ (nm)	λ_{em} (nm)
DBDNC-NPs – PSMA (0.05:1)	635	645
DBDNC-NPs – PSMA (0.2:1)	635	645
DBDNC-NPs – PSMA (0.5:1)	638	711

Figure S3. Comparison of the normalized (a) absorption and (b) fluorescence spectra of DBDNC-NPs with various mass ratios of DBDNC to PSMA in water. λ_{ex} = 580 nm for recording emission spectrum. (c) Summarized parameters of the excitation and emission maxima of DBDNC-NPs with various mass ratios of DBDNC to PSMA.

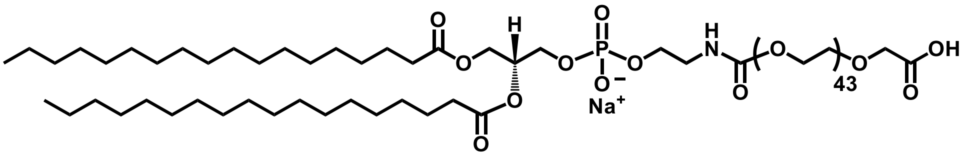

Figure S4. Chemical structure of DSPE-PEG2000-COOH.

Table S1. Summarized PLQY of the deoxygenated NPs water dispersions. Excitation wavelength: 360 nm.

**NPs**	DBDNC-NPs-DSPE (0.5:1)	DBDNC-NPs-PSMA (0.5:1)	DBDNC-NPs-PSMA (0.2:1)	DBDNC-NPs-PSMA (0.05:1)
PLQY	2%	1%	1%	7%

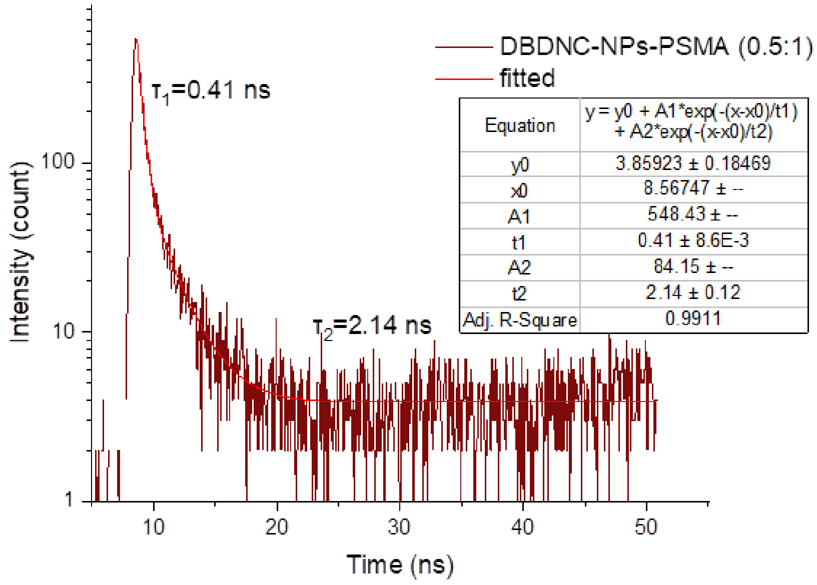

Figure S5. The emission decays and fitting of DBDNC-NPs-PSMA (0.5:1) in water dispersion at room temperature. Excitation wavelength: 580 nm. Integrated wavelength range for 600-825 nm.

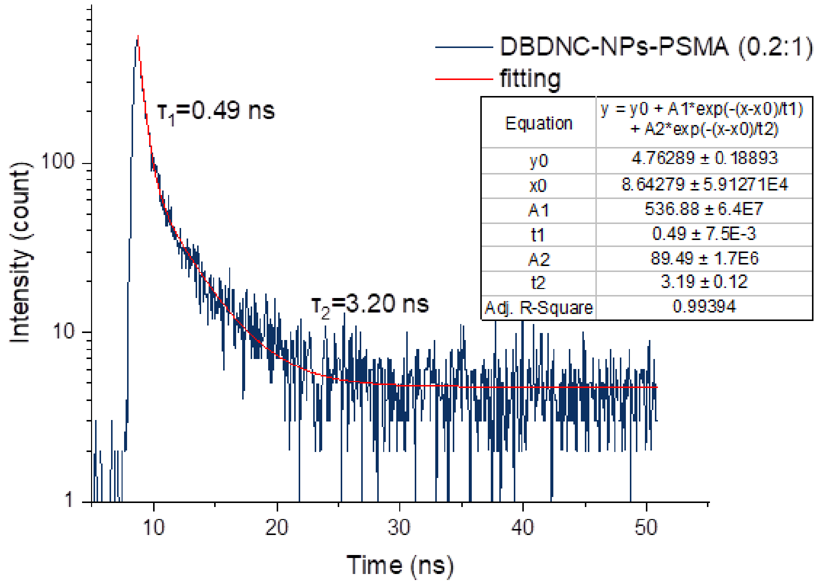

Figure S6. The emission decays and fitting of DBDNC-NPs-PSMA (0.2:1) in water dispersion at room temperature. Excitation wavelength: 580 nm. Integrated wavelength range for 600-825 nm.

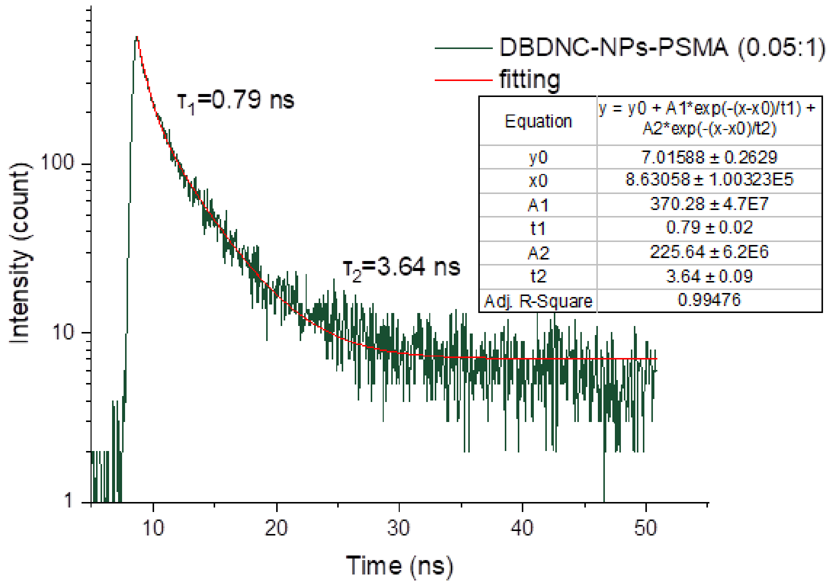

Figure S7. The emission decays and fitting of DBDNC-NPs-PSMA (0.05:1) in water dispersion at room temperature. Excitation wavelength: 580 nm. Integrated wavelength range for 600-825 nm.

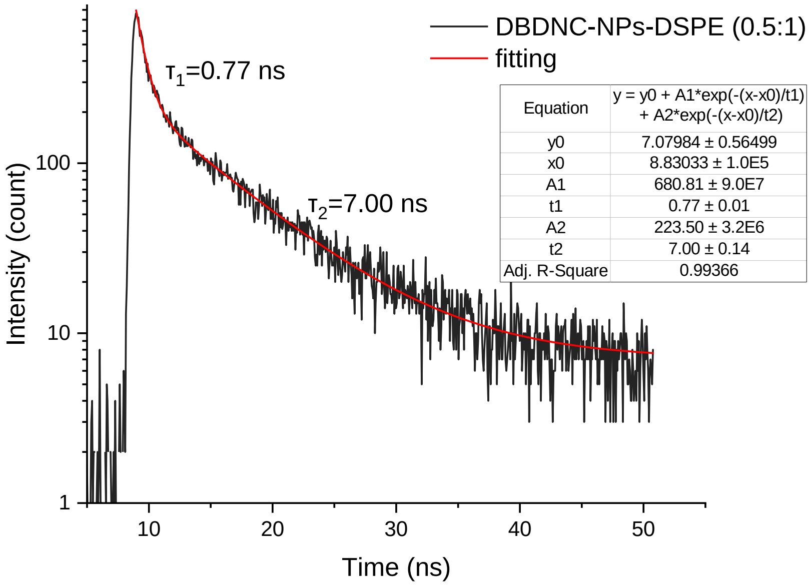

Figure S8. The emission decays and fitting of DBDNC-NPs-DSPE (0.5:1) in water dispersion at room temperature. Excitation wavelength: 580 nm. Integrated wavelength range for 600-825 nm.

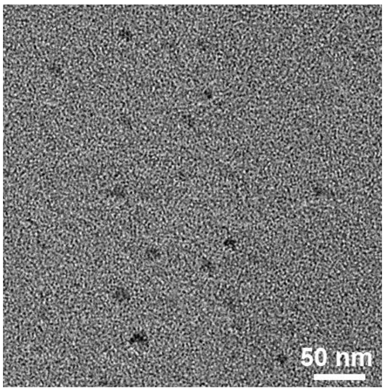

Figure S9. TEM image of the prepared DBDNC-NPs-PSMA (0.5:1) (25 μg mL^{-1}, based on the mass of DBDNC without PSMA). Scale bar: 50 nm.

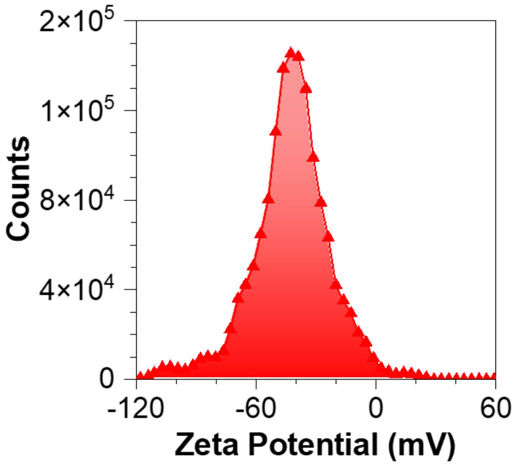

Figure S10. Zeta potential of DBDNC-NPs-PSMA (0.5:1) in water (25 μg mL^{-1}, based on the mass of DBDNC without PSMA).

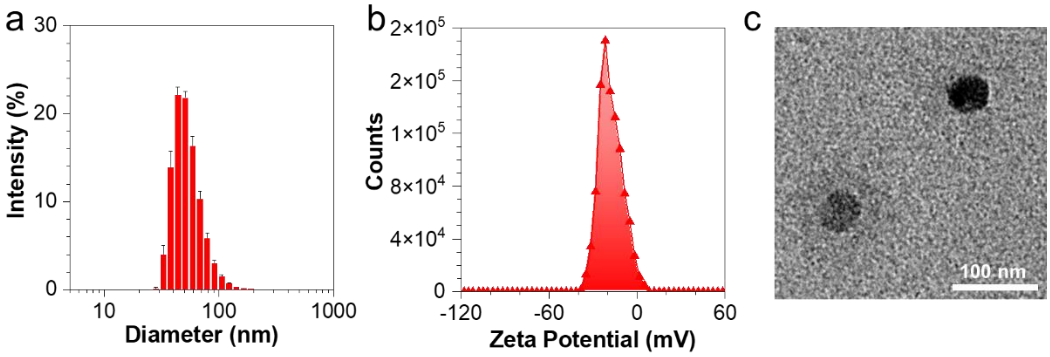

Figure S11. (a) Size distribution of DBDNC-NPs-DSPE (0.5:1) by DLS analysis. (b) Zeta potential of DBDNC-NPs-DSPE (0.5:1) in water. (c) TEM image of DBDNC-NPs-DSPE (0.5:1). Scale bar: 100 nm. The concentration of NPs was 25 μg mL^{-1}, based on the weight of DBDNC without DSPE-PEG2000-COOH.

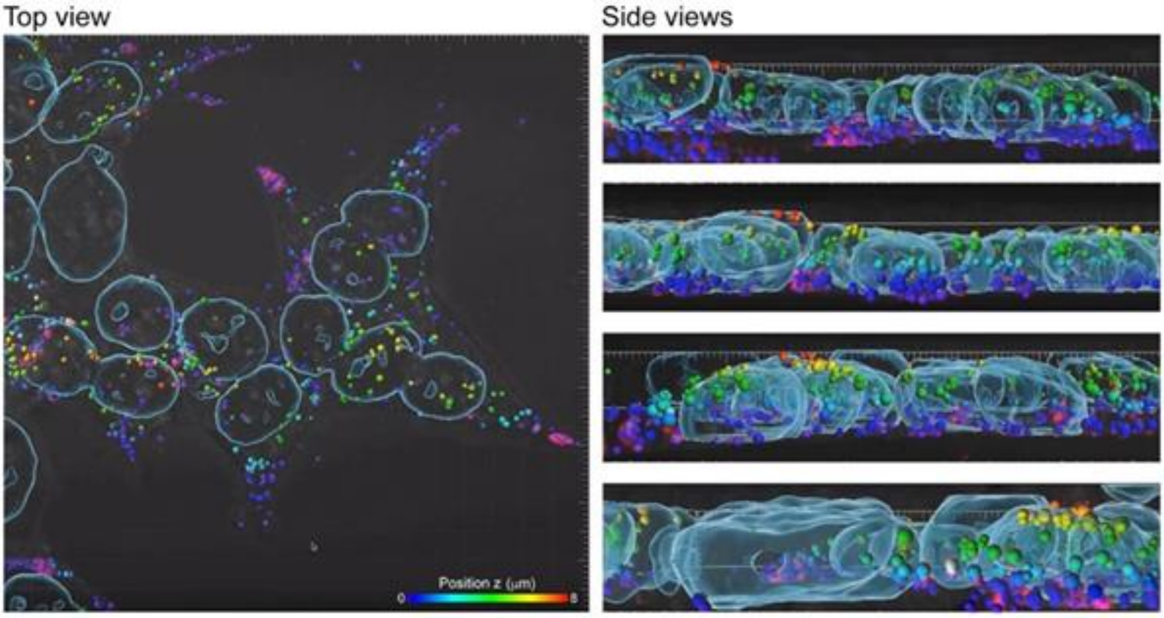

Figure S12. Representative 3D image of DBDNC-NPs loaded into HEK293T cells. The NPs are color coded according to their z-position (depth). Color-coded bar: 0 µm (blue) to 8 µm (red).

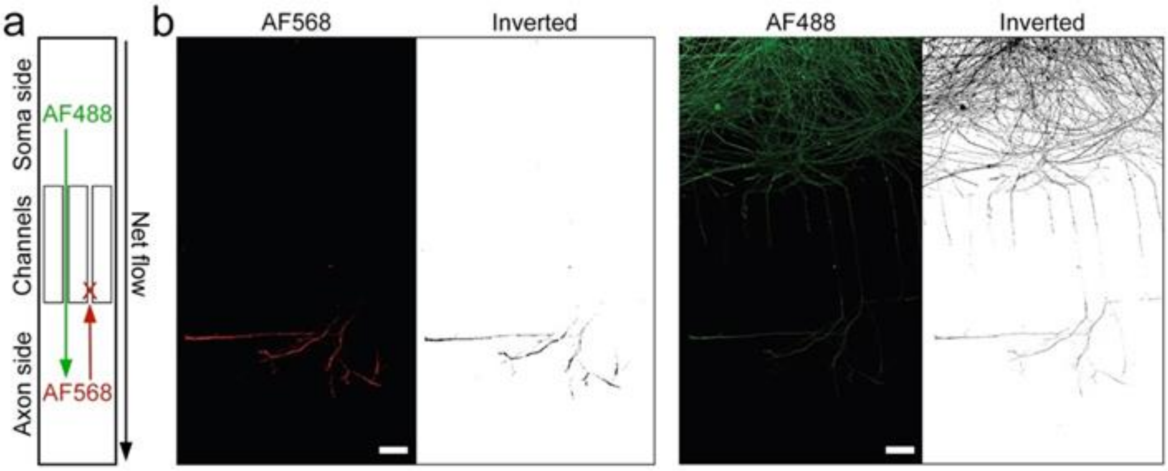

Figure S13. Fluidic isolation assay in MFC cultures. (a) Schematic representation of the assay. After labelling the soma and axon side with anti-β3-tubulin antibody, different fluorescent secondary antibodies were loaded into the soma side (AFF488) or into the axon side (AF568). As detailed in the Methods section, a net flow of medium was created between the soma side and the axon side, maintaining fluidic isolation of the neuronal cell bodies from their axons. (b) Representative confocal images of β3-tubulin labelling with AF568 restricted to the axon side only and with AF488 in both soma and axon sides of the MFC. Scale bar: 100 µm.

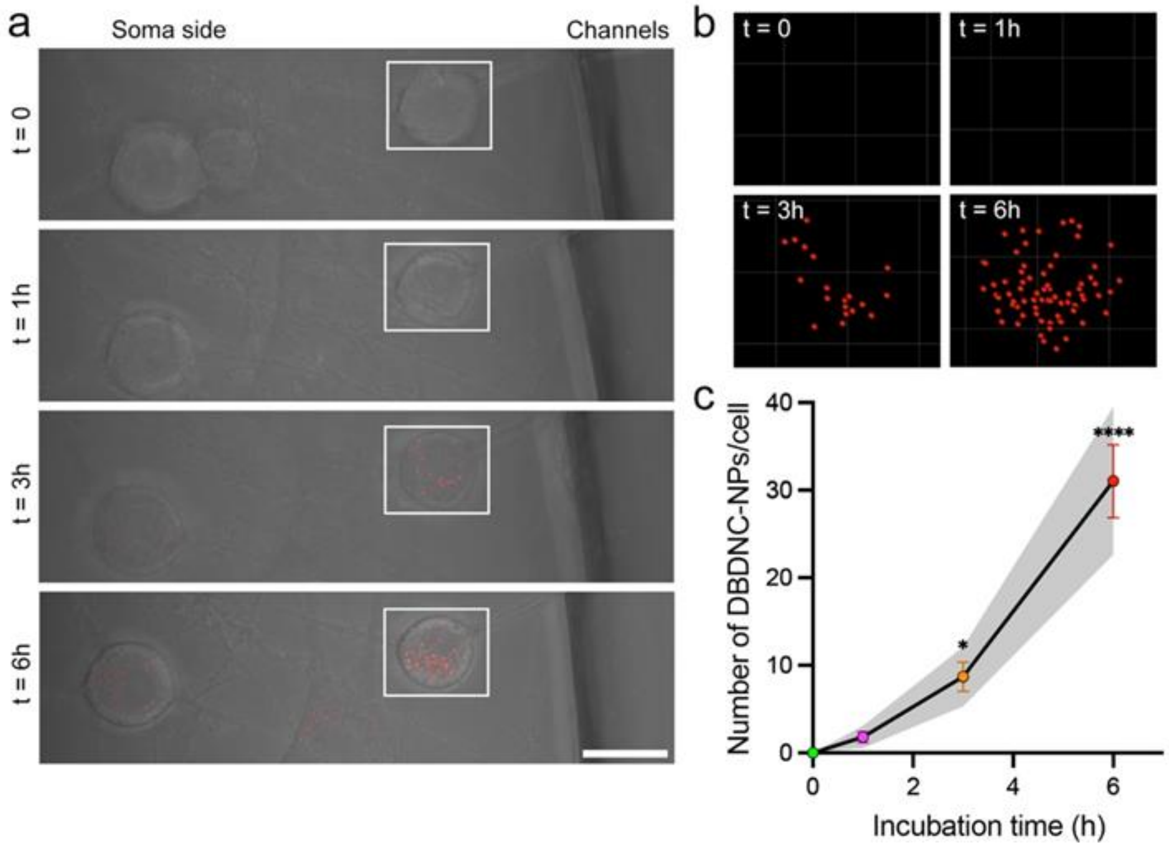

Figure S14. Somatic accumulation of DBDNC-NPs in MFC-cultured neurons. (a) CLSM time-series images of DBDNC-NPs (red) accumulating in neuronal soma from 0 to 6 hours of incubation of NPs in the axon side of the MFC. "Time = 0" is the initial time of incubation. Scale bar: 20 mm. (b) Corresponding 3D spot rendering of DBDNC-NPs in the soma boxed in (a) at different time point. (c) Quantification of the number of DBDNC-NPs in neuronal soma over time. Data shown as mean ± s.e.m. (n = 40 cells from 3 MFC cultures obtained from 1 mouse) and 95 % confidence interval (CI; shaded area). One-way ANOVA *: $p = 0.0189$, ****: $p < 0.0001$).
